# An interview with Patrick O Brown on the origins and future of open access

**DOI:** 10.1186/1741-7007-11-33

**Published:** 2013-04-15

**Authors:** Patrick O Brown

**Affiliations:** 1 Department of Biochemistry, Stanford University School of Medicine, Stanford, California, USA; 2 Howard Hughes Medical Institute, Stanford University School of Medicine, Stanford, California, USA

## 

Patrick O Brown, known as Pat, did his undergraduate and medical training at the University of Chicago, where he also studied under Nicholas Cozzarelli for his PhD. He was a paediatrics resident at Children's Memorial Hospital, before taking a postdoctoral research position with Michael Bishop and Harold Varmus at the University of California at San Francisco. He subsequently moved to Stanford where he played an instrumental part in the development of microarray technology and its applications and where he is now a Professor of Biochemistry and Howard Hughes Medical Institute investigator.

**Figure:**
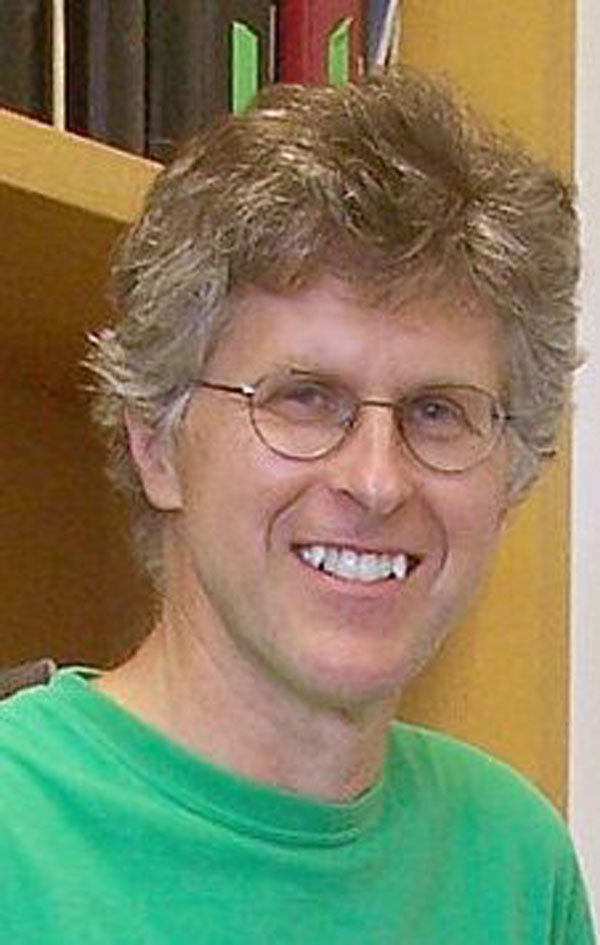
**Patrick O Brown**.

Pat has a long association with BioMed Central, dating from its origins at the beginning of the open access movement, and is on the Editorial Board of *BMC Biology*; so we asked him to give his own perspective on open access and how it began.

## Do you remember how and when the idea of open access first occurred to you?

It occurred to me in a kind of a roundabout way. I had been working on studies that involved looking at the expression of thousands or tens of thousands of genes under hundreds of different physiological conditions and in different developmental states, mostly in yeast, some in humans, so we had in our hands data sets that consisted of millions of quantitative measurements of gene expression that you can think of as a map that relates physiology and development and disease to patterns of expression of thousands or tens of thousands of genes.

## This was in the very early years of microarrays?

Yes. Some of that data is interpretable without any external information, but its value is hugely enhanced if we can use it as a way of linking on the one hand things that we already know about physiological systems or developmental systems or diseases or particular tissue cell types, and on the other hand whatever we already know about the genes or their products: the links that are provided by those millions of data points in the gene expression arrays add a huge amount of value to existing information.

So every time we did an experiment my colleagues and I found ourselves digging up hundreds of papers to educate ourselves about things that we were seeing in the data. By that time a substantial fraction of the journals that were most relevant to what we were doing were being published online and since we were at Stanford we had access to at least most of them, but we knew we were not getting full value out of our data, first because we still didn't have ready access to all the available knowledge that was published somewhere and would enable us to make better sense of it. Equally importantly, the manual search for published information that could add value to our data was unscalable. So we wanted to put the entire corpus of relevant articles in a database that would let us automate the process, but we were thwarted by publishers who strictly forbade downloading and automated analysis.

That led me to ask why should publishers be able to control what I can do with information that was published by my scientific colleagues whose motivation was exactly to have their discoveries contribute to future discoveries? And it became obvious that there were things about the way the scientific literature was organized that were anachronistic in 1997 when we had already existing tools that we could use to so to speak hyperlink things so that you could reorganize information in systematic ways, but they weren't really being exploited by the conventional scientific literature.

Then at about that time my lab had published a paper that involved a lot of supplementary information that we posted online the main server at Stanford, which was used by maybe a hundred scientists at Stanford, and more than half of all the bandwith of the server was taken up by people downloading our data. And so I thought OK well actually we should just stop publishing in journals altogether, and when we have something to report we'll just post it up on this server and spread the word and bypass the whole annoying experience of publishing in journals, and just let the world decide if what we have is interesting. Well that was kind of a primitive idea but...

## This is something that physicists were already doing

I was just about to say that. At the time I wasn't aware of what was then called the XXX.LANL preprint server [now http://arXiv.org] that Paul Ginsparg had created, but it was very close to that kind of thing that I had in mind. Paul had started a little server as an informal mechanism for exchanging preprints in his small community that just grew organically because it was such a useful thing.

## So your idea was that there was no reason why this couldn't be extended to biological sciences?

Yes. The way the existing journal system operated was completely unsuited to very data intensive research where the real need is to be able to use large systematic datasets as a way of providing links between disparate bits of information.

## And it was about that time that you started talking to people, including Vitek Tracz, who founded BioMed Central, about how to change the publishing world

Yes. I don't remember the exact timing, but I do remember talking relatively early on in the process with Vitek and also with David [Lipman], and a few months later when I was organizing a genomics meeting with Gerry Rubin at Banbury [the Banbury Center at Cold Spring Harbor] I set up a session to which I invited David and Paul Ginsparg - because they hadn't met - with the intention to brainstorm about how we could transform scientific publishing.

Then around the same time Harold Varmus, who was then Director of the NIH - he was a friend of mine because he'd been my postdoc advisor some years before, and a fellow non-conformist personality was coming out to San Francisco, so I met him at a coffee shop, and went through the idea of setting up a NIH hosted server where scientists could post their work when they felt it was ready to share with the world, and where it would be organized in a systematic way. David Lipman and I had already started having conversations about things that still actually haven't come to pass, discussing how one could make the system work. I still think they're good ideas, but...anyway, the upshot of it was that Harold was quite receptive and organized a follow-on meeting at NIH, and there was a lot of enthusiastic support, and from people of all stripes: it wasn't just a bunch of genome geeks and bioinformatic types.

And then over the next month or so Harold and I were bouncing back and forth by e-mail some documents about how this system might operate and what it could try to accomplish, and then Harold sent around to some publishers and societies, and also posted on the Director's website a proposal for something that was dubbed e-Biomed. It's still up there, at http://www.nih.gov/about/director/pubmedcentral/ebiomedarch.htm.

## It is still up there, and what it makes clear is that once the idea of open access started to be developed, it was very much in the model of subscription-based print publishing, with peer review and so forth, which was not what you originally had in mind

Absolutely not. What we had in mind originally was much more radical in every way. And I should say those of us who have been involved in this for some time have absolutely not given up on this, it's just been more slow and incremental than we had wished.

## But what about open access itself - doesn't it seem that open access has gained ground so fast that that battle is now all but won?

Well let's put it this way: I think the tipping point was reached quite a while ago, and so in that sense it's won: I think that there's absolutely no going back: there's a relentless ratchet effect so that step after step after step the subscription business model is becoming less and less sustainable, and the notion of open access publishing as the only right way to do things is becoming pretty well established I would say in the scientific community, and I think most of the existing publishers recognize that.

## So we're left with the issue of how to escape from the current peer review model - do you see that as the next frontier?

The thing that is most sick right now, besides the continued existence of restricted access, is the delay between an important discovery or idea or publishable body of information and its actual publication. For example, when a scientist makes an observation that's the genesis of a new way to treat an important disease and submits it to a journal, nobody sees that paper until nine months later. Nine months later someone sees it, takes the next step, submits their own paper and it finally comes out another nine months later. Do a few cycles of that and something that, let's say, can save millions of lives actually becomes available two years later than it had to, entirely because of a delay between a discovery being made and its being shared with the world. And that's the situation we're in now. That delay is the opportunity cost we pay for requiring peer-review before publication. I'm all for peer review, but I'm not for way it's done now. You could have the peer review process - or a better peer review process - AFTER a paper has been published.

But let me just take a step back and say one thing: when we first started thinking about open access there were a lot ideas about how to fix scientific publishing, but we all realized quickly that by far the most important was open access. That was the *sine qua non *for everything else we wanted - to have the scientific literature be available unrestricted, to anyone to use in any way they wanted. Once you have that, then you not only make the benefits of the published research available to the whole world, but you have the potential for all kinds of experimentation, for building new tools, news ways of structuring the information, and so on.

## Do you think that has yet been fully exploited?

It's not fully exploited, though I think it's almost at the point where it will be, because for people to invest in building new systems for organizing and navigating the information you have to have a critical mass of raw material. You can't have a patchwork. And I think the open access literature at this point is approaching a critical mass where that body of information is enough to allow us to ignore all the non-open access literature. We'll reach a point where there's a positive feedback loop: with enough information, people will develop tools to make open-access information more valuable, and then it will become increasingly more valuable than the restricted-access information because it's not only accessible but it's had value added to it in ways that can't be applied to the restricted-access material, so that non-open access becomes progressively marginalized, and nobody wants to be in that space.

## Note

This article is part of the *BMC Biology *tenth anniversary series. Other articles in this series can be found at http://www.biomedcentral.com/bmcbiol/series/tenthanniversary.

